# The Apoe^−/−^ mouse model: a suitable model to study cardiovascular and respiratory diseases in the context of cigarette smoke exposure and harm reduction

**DOI:** 10.1186/s12967-016-0901-1

**Published:** 2016-05-20

**Authors:** Giuseppe Lo Sasso, Walter K. Schlage, Stéphanie Boué, Emilija Veljkovic, Manuel C. Peitsch, Julia Hoeng

**Affiliations:** Philip Morris International R&D, Philip Morris Products S.A. (Part of Philip Morris International Group of Companies), Quai Jeanrenaud 5, 2000 Neuchâtel, Switzerland; Max-Baermann-Str.21, 51429 Bergisch Gladbach, Germany

**Keywords:** Apoe, Lipoprotein, Mouse model, Atherosclerosis, Emphysema, COPD, Cigarette smoke, Tobacco heating system

## Abstract

Atherosclerosis-prone apolipoprotein E-deficient (Apoe^−/−^) mice display poor lipoprotein clearance with subsequent accumulation of cholesterol ester-enriched particles in the blood, which promote the development of atherosclerotic plaques. Therefore, the Apoe^−/−^ mouse model is well established for the study of human atherosclerosis. The systemic proinflammatory status of Apoe^−/−^ mice also makes them good candidates for studying chronic obstructive pulmonary disease, characterized by pulmonary inflammation, airway obstruction, and emphysema, and which shares several risk factors with cardiovascular diseases, including smoking. Herein, we review the results from published studies using Apoe^−/−^ mice, with a particular focus on work conducted in the context of cigarette smoke inhalation studies. The findings from these studies highlight the suitability of this animal model for researching the effects of cigarette smoking on atherosclerosis and emphysema.

## Overview

Atherosclerosis is a chronic disease in which systemic inflammation underlies the accumulation of plaques in the arterial intima. Plaques have a lipid-rich core and a thin fibrous cap that may rupture, causing thrombosis of the narrowed vessels [1]. If not properly controlled, atherosclerotic plaques can obstruct the arterial lumen, leading to cardiovascular diseases (CVDs), such as coronary heart disease (CHD), heart attack, stroke, or angina. More recently, an additional mechanism has been recognized, and it is responsible for fatal vascular occlusions predominantly in younger and female patients [2]. Indeed, thromboses can also arise from superficial erosion sites in vascular walls characterized by endothelial cell apoptosis on an underlying lipid-poor but proteoglycan- and glycosaminoglycan-rich plaque structure. It is important to highlight that statin treatment, lifestyle changes and other factors may favor the stabilization of the classical lipid-rich plaques [2–4]. Nevertheless, in 2011, CHD was responsible for around one in every seven deaths in the United States [5].

The development of CVD is associated with a number of risk factors, including smoking, physical inactivity, obesity, high blood pressure, diabetes, dyslipidemia, and genetic factors. Therapeutics targeted reducing CVD face a difficult pre-clinical hurdle because of a dearth of appropriate animal models that can capture the complexity of the human disease, which may take over 50 years to result in any clinically apparent event.

The development of animal models for CVD has increased our understanding of its pathophysiology, and allowed researchers to assess not only the etiologic effects of diet and environmental factors but also to evaluate the effectiveness of potential therapies [6]. The most widely used murine models for atherosclerosis are apolipoprotein E (Apoe) knockout (Apoe^−/−^) and low density lipoprotein (LDL) receptor deficient mice (Ldlr^−/−^), which both develop hypercholesterolemia [7–9]. The two models have both advantages and disadvantages depending on the goals of study. In fact, on a chow diet, Apoe^−/−^ mice show higher plasma total cholesterol level compared with Ldlr^−/−^, and thus, develop severe atherosclerotic lesions as soon as a few weeks after birth [8, 10]. Moreover, the deficiency of the endogenous Apoe expression leads to an imbalance of cholesterol loading specifically in the macrophages. In turn, this stimulates cytokine and protease secretion, and triggers subsequent inflammation and extracellular matrix degradation [11]. These peculiar side effects related with the deficiency of Apoe but not Ldlr, confer to Apoe^−/−^ mice greater adaptability for studying several other diseases associated with inflammation and extracellular matrix degradation [12], such as Alzheimer’s [13, 14], erectile dysfunction [15], diet-induced steatohepatitis [16, 17], and recently also chronic obstructive pulmonary disease [18–21]. Other less frequently reported mouse models of atherosclerosis leverage alternative mechanisms of perturbing lipid metabolism. The hyperlipidemic APOE*3-Leiden mice express a human APOE isoform [22], and are described in greater detail below. In the scavenger receptor B1 (Scarb1) knockout mice, the atheroprotective HDL-C levels are elevated but—(apparently) paradoxically—atherosclerosis is increased [23, 24]. However, it has recently been shown that patients with a rare genetic inactivation of the SCARB1 also develop high levels of HDL-C associated with a higher atherosclerosis risk [24]. Double knockout mice for Scarb1 and Ldlr show more severe atherosclerosis and higher mortality than the single knockouts, particularly in combination with high fat diet [25]. For these mouse models of atherosclerosis, to the best of our knowledge, no smoke exposure studies have been reported in the literature.

The Apoe^−/−^ model initiated a new era for CVD research. The Apoe^−/−^ mouse was developed in 1992 by homologous recombination of embryonic stem cells, and it is currently the most widely used pre-clinical model of atherosclerosis [9, 26]. Apoe is a ligand for lipoprotein receptors involved in lipoprotein recognition and clearance. In particular, Apoe serves as a ligand that mediates the uptake of chylomicrons, very low-density lipoprotein (VLDL) and their remnants to hepatic receptors (LDL receptor and LDL receptor-related protein). Therefore, Apoe^−/−^ mice show delayed lipoprotein clearance and consequently develop hyper- and dyslipoproteinemia [27], severe hypercholesterolemia, and atherosclerotic lesions even when on a normal diet. Arterial fat deposits are observed as early as 3 months after birth [28, 29]. By 8 months of age, the coronary arteries can be almost occluded when streptozotocin treatment is applied for additional acceleration [30, 31].

In the context of CVD development, the creation of genetically engineered mouse models became necessary also because of differences in the lipid homeostasis between human and murine organisms. For example, plasma cholesterol in wild-type mice on a regular chow diet is ~ 80 mg/dl, and it is primarily carried by high density lipoprotein (HDL) particles; moreover, these mice present small amounts of LDL and other atherogenic lipoproteins, such as VLDL remnants. This high HDL/LDL ratio is maintained even when mice are fed with high fat diet, indicating that wild-type mice have a high resistance to atherosclerosis development. In contrast, human lipid profiles show the majority of cholesterol in LDL particles (110 mg/dl) [32]. These characteristics make humans susceptible to HDL/LDL cholesterol ratio fluctuation, conferring a high risk of atherosclerosis and subsequent CVD. However, cholesterol transport and metabolism are sufficiently similar in the two species, suggesting that induced disturbances in plasma lipoprotein metabolism through gene manipulation would also lead to atherosclerosis in mice. Thus, the atherosclerotic lesions in Apoe^−/−^ mice resemble human lesions in their sites of predilection and progression to the fibroproliferative stage [33, 34].

**Fig. 1:**
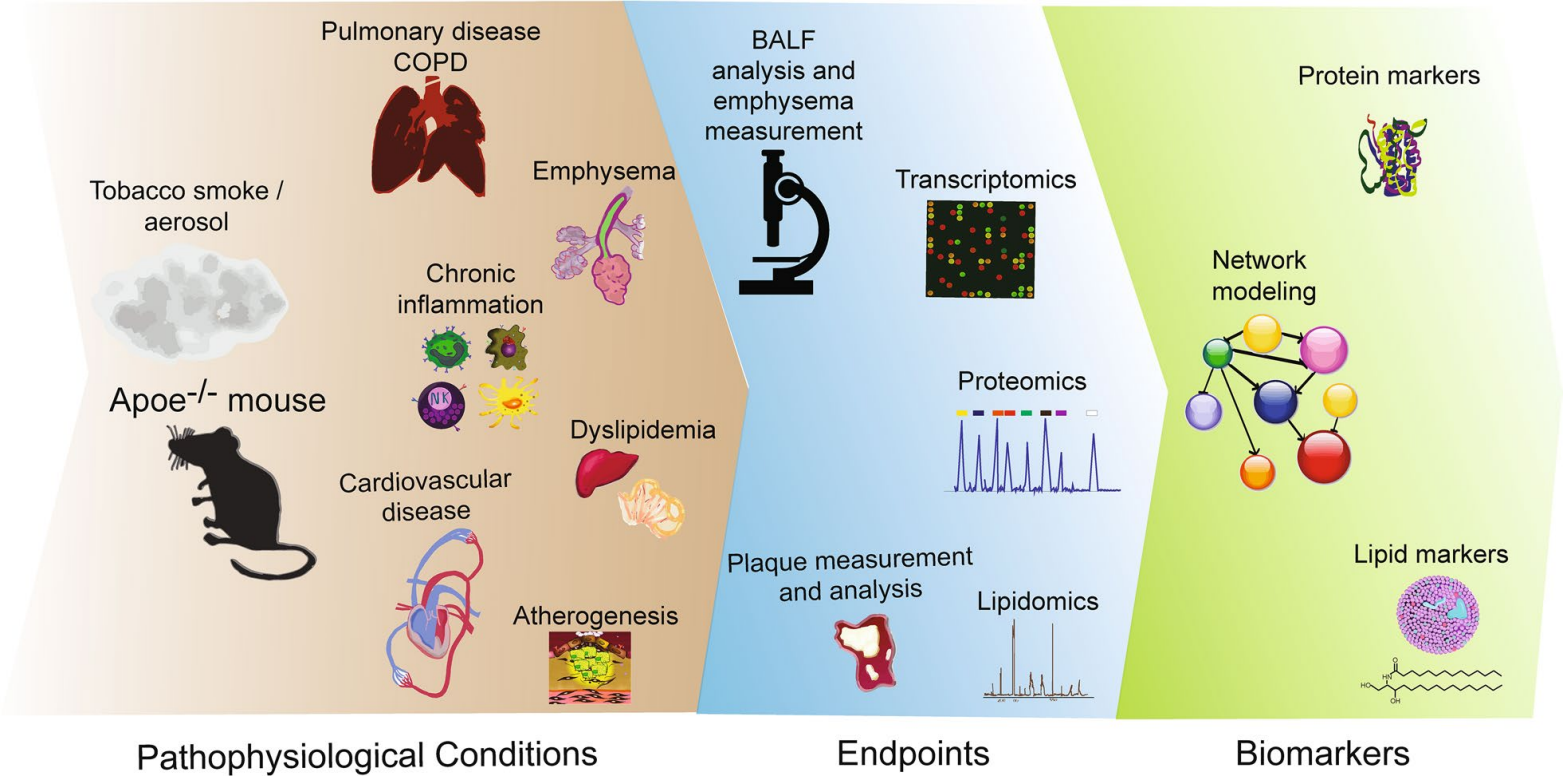
Overview of the pathophysiological events and endpoints in the context of inhalation toxicology studies in Apoe^−/−^ mouse model. *BALF* *b*roncho-*a*lveolar *l*avage *f*luid. *COPD* *c*hronic *o*bstructive *p*ulmonary *d*isease

This review aims to highlight the usefulness of Apoe^−/−^ mouse model in the context of inhalation toxicology studies addressing questions related to the role of cigarette smoke (CS), cessation, and candidate modified risk tobacco products (cMRTPs) [35] in the development of atherosclerosis and pulmonary diseases (Fig. 1).

## Chronic obstructive pulmonary disease and its association with CVD

Chronic obstructive pulmonary disease (COPD) is typically considered to be a progressive lung disease, although it is increasingly thought to be a generalized disorder with a wide range of phenotypes [36]. COPD also shares some risk factors with CVD, including cigarette smoking.

Growing evidence suggests that, as in CVD, chronic systemic inflammation is an important factor in the development of COPD. Smoking causes, among other effects, a systemic inflammation, which may explain why smokers can develop both diseases, as well as other comorbidities such as cancer. Generally, COPD patients have an increased risk of developing CVD, even after correcting for risk factors such as smoking, and most COPD patients die from CVD or cancer rather than respiratory symptoms [37]. Despite their similarities, these disorders are often studied in isolation and the increased risk of CVD in COPD patients is often underestimated [36]. As interactions between the two diseases are not accounted for, the treatment of patients with both COPD and CVD is therefore often suboptimal [38].

Most animal models show resistance to the development of CS inhalation-related pathologies. This is why animal models with functional deficits are used to study the effects of CS [39]. The study of multiple disease endpoints in one mouse model would be very advantageous, as seen in a study of hyperlipidemic APOE*3-Leiden mice [22], which are prone to atherosclerosis [21]. These mice were intranasally administered low-dose lipopolysaccharide following the induction of emphysema by porcine pancreatic elastase. They exhibited pulmonary inflammation [increased interleukin (IL)-6 concentrations] and increased atherosclerotic lesion areas, but the presence of emphysema did not appear to accelerate atherosclerosis. This suggested that CVD/COPD therapy should aim to lower pulmonary and systemic inflammation, rather than simply treating emphysema.

**Fig. 2:**
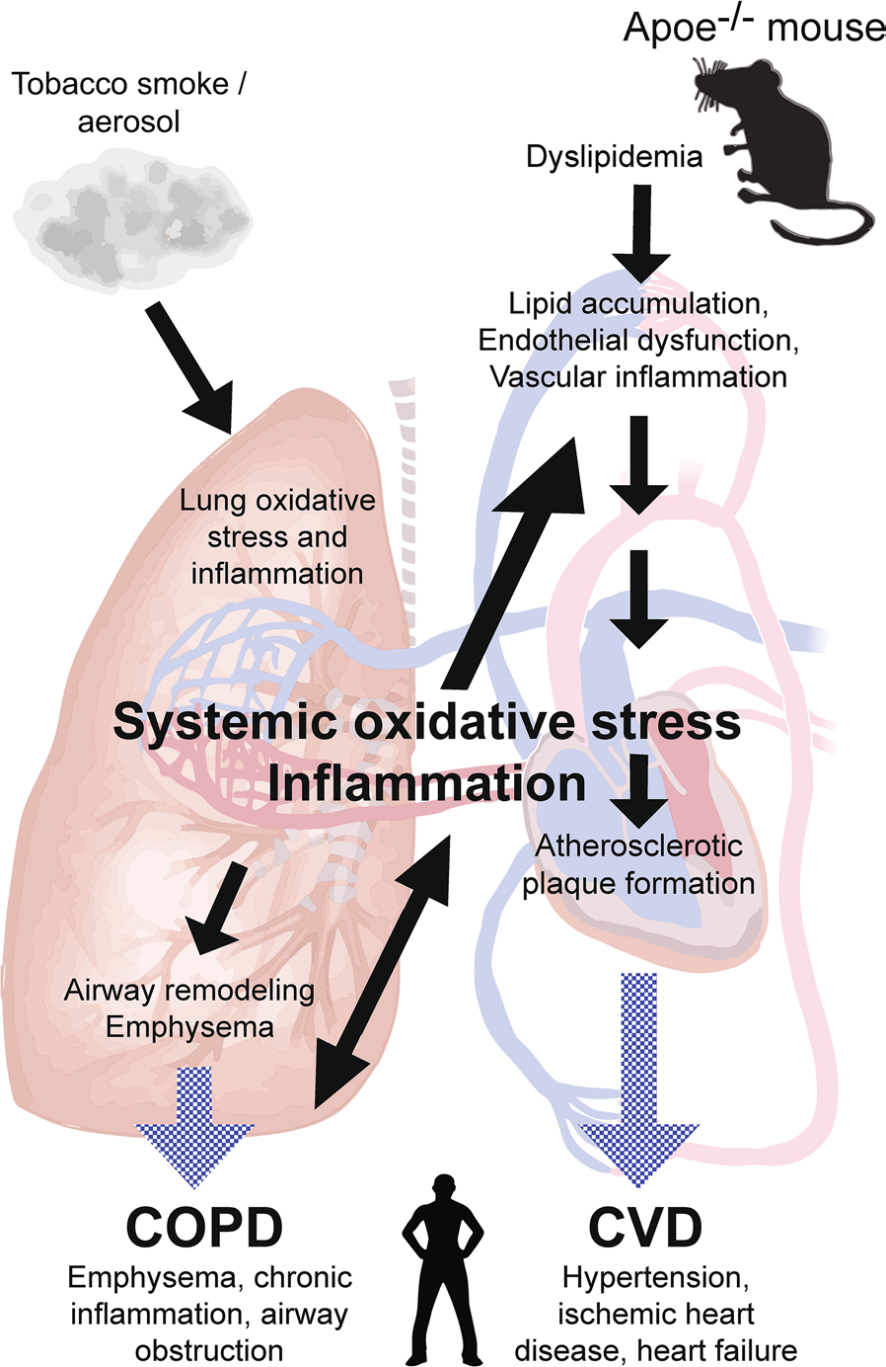
Lipid imbalance together with endothelial dysfunction and systemic inflammation determine the development of atherosclerosis as well as lung inflammation in Apoe^−/−^ mice (and humans). CS exposure enhances these processes, thereby increasing oxidative stress and inflammation processes

Apoe^−/−^ mice are a particularly useful strain to investigate comorbidities associated with cigarette smoking because, together with premature atherosclerosis [10], they show impaired alveologenesis [40] and develop emphysema [20, 41] (Fig. 2). In Apoe^−/−^ mice fed a Western-type diet, severe systemic hypercholesterolemia accompanied by the abnormal cholesterol efflux induced pulmonary inflammation through a TLR4/inflammatory/MMP cascade, all of which ultimately result in emphysema development [20]. CS exposure of Apoe^−/−^ mice clearly boosted these processes. In fact, CS increases oxidative stress, mitochondrial damage, and reduces glutathione levels, which in turn trigger arterial thrombosis and modulates the size and composition of neointimal lesions and thickening [42–44].

In a recent study by Han and colleagues [21], the plasma cholesterol concentrations of Apoe^−/−^ and Ldlr^−/−^ mice responded differently to sidestream CS exposure, most probably because of the differences in lipoprotein metabolism between the two strains [45]. Those findings highlighted the valuable knowledge gained from studying two smoke-related pathologies in one animal model, but our recent literature searches on PubMed did not retrieve additional studies investigating CS exposure effects on Ldlr^-/-^ mice.

The observed link between peripheral systemic changes in lipid metabolism and lung dysfunction seems to be a novel and clinically relevant factor in public health. As discussed above, it is already well established that patients with COPD have increased cardiovascular morbidity and mortality, but recent observations in both experimental models and humans have indicated that the reverse is also true, with the development of COPD in patients with atherosclerosis [46].

## Human APOE polymorphism and CS: possible association with increased CVD risk

Diseases such as CVD and CHD are the result of physiological system homeostasis imbalance. Genetic (e.g., gene expression), environmental factors (e.g., smoking), or the interaction thereof may be the leading cause of these dangerous imbalances. Functional gene polymorphisms account for much of the different responses of human beings to environmental changes, increasing (or reducing) the risk of disease development.

In the context of CVD and APOE genotype, human populations show a very low frequency of APOE gene deficiencies. However, APOE is polymorphic in humans and plasma LDL cholesterol levels, hence atherosclerosis risks are strongly associated with the three common APOE isoforms, in the following order: APOE4, APOE3, and APOE2 [47]. Human APOE is a 299 amino acid protein whose isoforms are encoded by three APOE alleles (ε2, ε3, ε4) that give rise to six different genotypes (ε2/2, ε2/3, ε2/4 ε3/3, ε3/4 and ε4/4) [48]. The association between the three isoforms and the plasma cholesterol levels is attributed to the different affinity with the LDL receptor [49], secondary to the recognition and internalization of the APOE containing particles [50–52]. Studies on the ε2 allele showed contrasting results. In fact, it was positively associated to hypertriglyceridemia [48], but also longevity [53] and negatively associated with myocardial infarction [54, 55]. APOE3 is the most common isoform, and it does not seem to be related to increased risk of CVD or other pathologies. On the contrary, the ε4 allele has been associated with CHD [47], Alzheimer’s disease [56], age-related cognitive decline [57], and other diseases [58]. Clinical studies conducted in the past 20 years showed not only the role of APOE polymorphisms on CVD occurrence, but also the interactions with environmental factors such as CS.

CS is associated with increased risk of CHD in men with all APOE genotypes, independently from the classical risk factors, including plasma lipid levels. The modification of the CHD risk associated with cigarette smoking by APOE polymorphism, was analyzed in the Northwick Park Heart Study II (NPHSII) [59], a prospective study of CHD in over 3000 UK men. In never-smokers, the CHD risk was similar in all APOE genotypes. The presence of the ε4 allele confined the CHD risk to current smokers alone and was independent of other classical CHD risk factors, including plasma lipid levels [60]. Interestingly in the ε4 ex-smokers, the risk decreased significantly, emphasizing the benefit of smoking cessation. The mechanism explaining this correlation could be detected in the higher levels of ROS in ε4 subjects [61]. Altogether, these studies highlighted the importance of the gene-environment interaction in the susceptibility to pathologies like CVD and CHD and its clear multifactorial nature.

The ability to study comorbidities in the Apoe^−/−^ mouse model fulfils the “Reduction” (minimize the number of animals used per experiment or study) and “Refinement” (minimize the pain, suffering, distress or lasting harm that may be experienced by the animals) points of the 3R principle in animal research [62, 63]. Of note, the reduction and refinement together with the “Replacement” principle (aiming in developing methods which avoid or replace the use of animals in research), are fundamental towards achieving a good match between preclinical study outcomes and clinical trials in human beings.

## Lipoprotein homeostasis in humans and mice

Lipoproteins are a complex and heterogeneous population of soluble macromolecular aggregates responsible for the transport of water-insoluble lipid molecules (mainly triglycerides, cholesterol, fatty acids, and phospholipids) from their site of synthesis (e.g., liver) or absorption from food (e.g., gut), to sites of use (e.g., muscle and adipose tissue). There are five types of lipoprotein particles (from smallest to largest): HDL, LDL, IDL, VLDL (high, low, intermediate, and very low-density lipoproteins), and chylomicrons. The lipid cores of these lipoprotein particles are coated by phospholipids and specific apolipoproteins that play a crucial role in many fundamental processes, such as efflux and transport [64], enzyme activation [65], and receptor binding [50, 51, 66].

Lipoprotein metabolism pathways differ according to the source of contained lipids and show species specificity that is important for preclinical studies.

*Exogenous lipoproteins* contain dietary fat (triglycerides and cholesterol) that is assembled in chylomicron particles [67]. Chylomicrons are released into the lacteal vessels, and at the capillaries in peripheral tissue as free fatty acids, transforming themselves into APOE-coated chylomicron remnants (CM). CM particles penetrate the fenestrated endothelium in the liver and will subsequently directly interact with ApoE-receptors, like the LDL receptor or the LDL receptor related protein 1 (LRP1) [68, 69]. The involvement of the LDL receptor in CM remnant clearance has been investigated showing contradictory results. In fact, although it accounts for the bulk clearance of serum lipoproteins into the liver, including the apoE-containing CM remnants, LDLR deficiency in both patients and animal models does not result in a defective clearance of CM remnants, indicating the existence of alternative APOE-specific remnant receptors for their clearance [70]. The LDL receptor related proteins might significantly contribute to the CM clearance. LRP1 belongs to the LRP family that represents a group of structurally related transmembrane proteins involved in a diverse range of biological activities [71]. The discovery that LRP1 binds APOE led to the notion that it acts as a remnant receptor. Consistently, LRP1 deficiency or inhibition does result in a decreased clearance of CM remnants.It is noteworthy that the metabolism of exogenous lipoproteins is preserved across species [72].

**Fig. 3:**
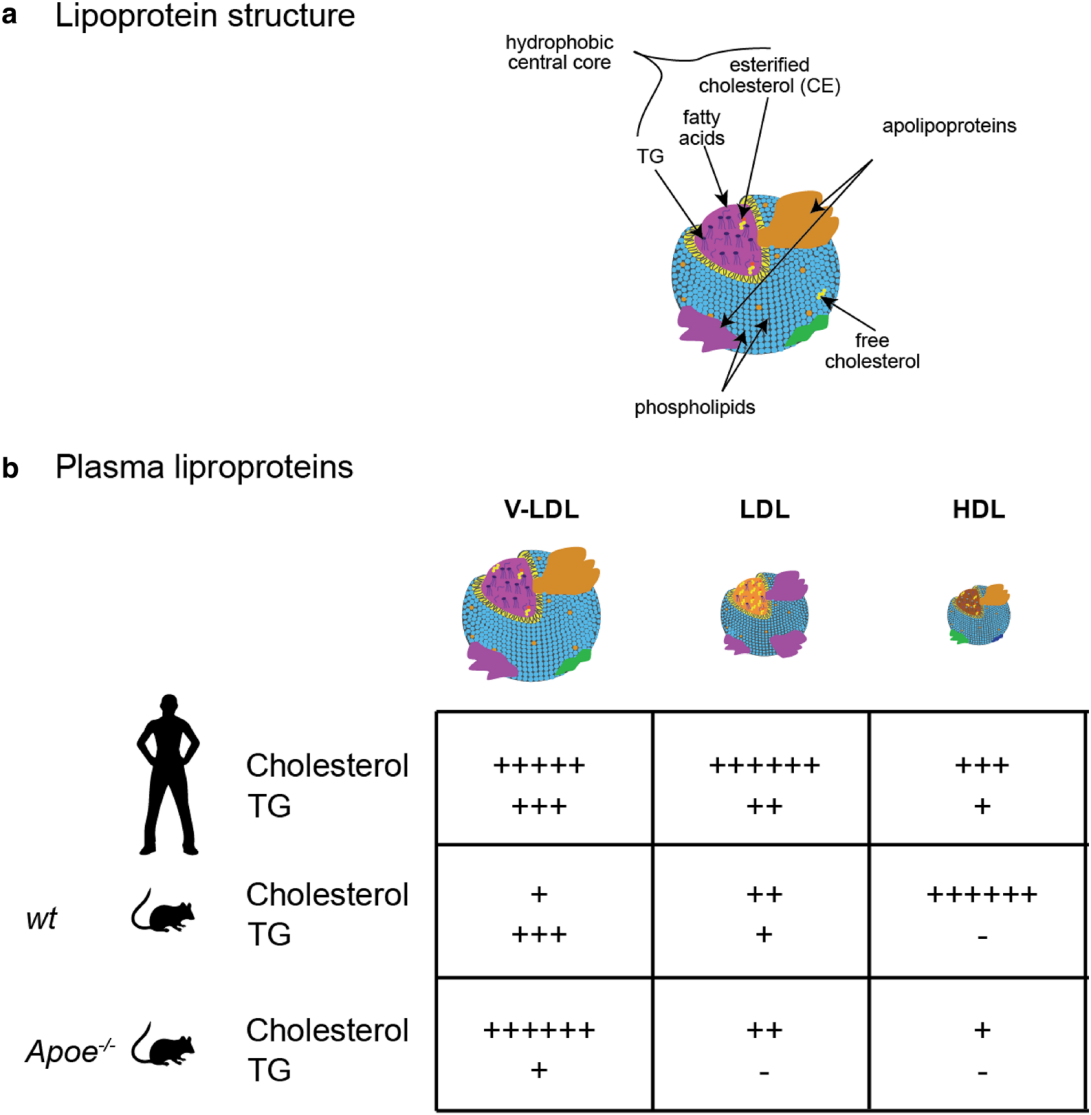
Lipoprotein structure and abundance in humans, wild type, and Apoe^−/−^ mice plasma. **a** The general structure of a lipoprotein includes a hydrophobic central core consisting of triglycerides, fatty acids, and esterified cholesterol, and a surface monolayer of phospholipid, free cholesterol, and specific apolipoproteins. **b** Schematic representation of cholesterol and triglycerides (TG) abundance in the different plasma lipoprotein species among humans, wild type, and Apoe^−/−^ mice

Conversely, the *endogenous lipoprotein* metabolism is associated with different key players between humans and mice, thus contributing to the missed overlap between lipid profiles of the two species under normal conditions (Fig. 3). Of note, ApoB is the main apolipoprotein of LDL, IDL and VLDL, fundamental particles in the endogenous lipid transport. Two molecular species of ApoB exist: ApoB100 is the full-length molecule that is synthesized in the liver, where VLDL particles are assembled for release into the circulation [73]. ApoB48 is produced by RNA editing from the same gene (APOB), and corresponds to the N-terminal fragment of ApoB100, therefore lacking the LDLR binding region. In mice this latter isoform is expressed in both the liver and in intestine, while in humans, it is only expressed in the intestine [74].

At the peripheral tissue capillaries, VLDL particles are transformed into IDL [68] after releasing free fatty acids. Part of the remaining VLDL particles and/or partially catalyzed VLDL particles bind to VLDL receptors expressed in peripheral tissue [75]. In mice, ApoB48-containing VLDLs and IDLs are internalized by the LRP8 expressed in the liver. Since the fractional catabolic rate for ApoB48-containing lipoproteins is very high compared with that of ApoB100-containing lipoproteins [76], VLDL and IDL almost disappear from mouse circulation, with the slightest contribution to LDL formation. LDL cholesterol thus appears very low in wild-type mice compared with humans (Fig. 3).

The second important difference between human and mouse endogenous lipoprotein distribution pertains to the HDL cholesterol that, contrarily to LDL, is very high in mice and low in humans. This difference is mainly due to the absence of the cholesterol ester transfer protein (CETP) in mouse plasma compared with the human one [77]. Thus, during the reverse cholesterol transport from peripheral tissue to the liver, HDL particles receive free cholesterol from macrophages through scavenger receptor type B-I (SR-BI) and ATP-binding Cassette proteins (ABCs), such as ABCA1 [78]. In humans, after lecithin: cholesterol acyltransferase (LCAT)-mediated esterification, the esterified cholesterol in HDL particles is transferred to VLDL, IDL, and LDL thanks to the CETP [79]. The absence of this enzyme in mice prevents the cholesterol depletion of HDLs that therefore, represent the major lipid-containing particle in the mouse plasma.

## Apoe^−/−^ model, CS, and atherosclerosis: an overview

Apoe^−/−^ mice have been used to study the effects of diverse risk factors on the development of atherosclerosis (Table 1). A long-term (12-month) study assessed the impact of mainstream smoke and a high-fat diet both separately and in combination [27]. It revealed that mainstream smoke significantly increased atherosclerotic plaque size in the brachiocephalic artery, while both risk factors combined increased the number of elastin-rich layers of the plaques, thus accelerating atherosclerosis.

**Table 1:** Functional endpoint categories related to atherogenesis in studies conducted in Apoe^−/−^ mice

Exposure category	Exposure route	Ref	Dyslipidemia	Inflammation	Thrombosis	Oxidative stress	Plaque development	Vascular function (constriction)	Vascular function (relaxation)	Vascular remodeling
Motor vehicle emissions	Intraperitoneal	[128]						↓→	↓→	
		[129]				→↗				
	Nasal	[130]	→	→↘			→↘↗			
		[131]					↑			
	Oropharyngeal aspiration	[132]					↑	→	→	
	Whole body inhalation exposure	[133]				↑↗		↑→↗		↑→↘↗
		[134]		↑→		↑→↗	↓→↘↗	↑→		↓↑→↘↗
		[93]					↑→↗	↗	→	
		[135]	→			↑	↑			
		[136]				↑	→	↓↑		
		[137]		↑→			↑	↓		
		[138]		↑→↗		↑→			↓→	↑↗
Environmental air particles	Intraperitoneal	[139]						→	↓↘	
	Oropharyngeal aspiration	[140]					↑			
	Whole body inhalation exposure	[141]					↑			
		[142]					↑	↓	↑	
		[143]					↑↗			
		[144]		↑			↑↗			↑↗
		[145]		↑		↑↑→	↑	↓		
		[93]					↑			
		[146]					↑↑	↗	→	
		[147]					↑			
		[148]	↑			↑				
		[149]				↑	↗			
		[150]					↑			
Motor vehicle emissions and environmental air particles	Whole body inhalation exposure	[151]		↗						
		[146]					↑↗	↗	→	
Environmental tobacco smoke surrogate (sidestream smoke)	Whole body inhalation exposure	[80]					↑→↗			
		[81]	→							
		[85]			↑					
		[21]	↑				↑			
		[91]				↑	↑			
		[152]	→				↑↗			
		[153]					↑			
		[93]					↑→↗			

Functional categories related to plaque development were created according to the specific endpoint investigated. This approach facilitated the analysis of dissimilar sets of data. Arrows indicate increase (upwards), decrease (downwards), no change (to the right), trend to increase (north east), and trend to decrease (south east) (Modified from [94])

Another study used an Apoe^−/−^ mouse model rather than larger animal models to provide more defined genetic background, greater statistical power in association with larger sample numbers, and more accurate lesion measurements [80]. The results showed that exposure to sidestream CS increased the size of atherosclerotic lesions, particularly in the thoracic region, and thus, the severity of atherosclerosis. This model was later used to test the effectiveness of dietary coenzyme Q, a lipid antioxidant, for preventing sidestream CS-induced formation of atherosclerotic plaques. Although coenzyme Q reduced the growth of spontaneous lesions, it did not significantly reduce the growth of smoke-induced plaques [81].

Atherosclerosis in the abdominal aorta can lead to the development of abdominal aortic aneurysms, which are reported to be about nine times more common in smokers than in non-smokers [82]. This process was investigated in an Apoe^−/−^ mouse model with angiotensin II-induced hypertension, which developed aneurysms in association with enhanced matrix metalloproteinase (MMP) gene expression [83]. Exposure to CS further upregulated *MMP* expression in the abdominal aorta, and exacerbated the development and severity of aneurysms.

To investigate the effects of CS exposure on plaque thrombogenicity, Apoe^−/−^ mice fed a high cholesterol diet were exposed to CS for 8 weeks (5 days/week). Following atherosclerotic plaque disruption, the tissue factor (TF) formed a complex with factors VII and VIIa during thrombin formation. Mice exposed to CS showed significantly greater TF immunoreactive areas than those exposed to filtered air, consistent with the TF immunoreactivity seen in human carotid plaques, albeit at lower levels [84]. Treatment with aspirin, an inhibitor of lipopolysaccharide-induced TF expression, attenuated the observed changes in TF in Apoe^−/−^ mice, which again paralleled the reduction in TF immunoreactive areas seen in smokers undergoing carotid endarterectomy who were treated with aspirin prior to surgery.

Thrombin formation activates the clotting cascade and platelet activity. Exposure to CS significantly increased platelet–fibrinogen binding in Apoe^−/−^ mice, leading to significantly greater thrombotic occlusion in these mice than in Apoe^−/−^ mice exposed to filtered air [85]. These changes were partially reversed by treatment with the Purinergic receptor P2Y, G-protein coupled, 12 (P2Y12) antagonist, cangrelor. Another P2Y12 antagonist, clopidogrel, also had positive clinical effects in human smokers [86], supporting the applicability of this mouse model to human studies.

The development of atherosclerosis involves a number of inflammatory and vascular remodeling events. The enzyme cyclooxygenase-2 (COX-2) is involved in prostaglandin biosynthesis and is overexpressed in atherosclerotic lesions. It can be activated by β-catenin, a component of cell–cell interactions. Apoe^−/−^ mice exposed to CS have been found to increase serum concentrations of the inflammatory cytokine interleukin-1, and enhanced translocation of β-catenin to the nucleus, leading to upregulation of COX-2 and inflammatory genes [87]. These cellular changes were proposed to underlie the proatherogenic effects in vascular tissue, while CS also disrupted the VE-cadherin–β-catenin complexes, induced phosphorylation events and increased vascular permeability [88].

The effects of CS exposure on immune suppression and subsequent atherosclerosis development were also examined in Apoe^−/−^ mice [89]. This study showed that intimal thickening induced by carotid arterial cuffing was greater in mice exposed to CS compared with mice exposed to filtered air. These effects of CS were associated with reduced anti-malondialdehyde oxidized LDL IgG titers. This immune modulation is thought to increase thickening of the vessel wall.

Some of the atherogenic effects of CS are mediated via oxidative stress. Systemic oxidative stress is increased in human smokers. This effect was modeled in a study using Apoe^−/−^ mice that reported significantly increased serum oxidative stress markers, including thiobarbituric acid-reactive substances, and oxidatively modified low-density lipoproteins. The serum and aortic 3-nitrotyrosine concentrations were also increased by exposure to CS, and these changes were alleviated by treatment with the antioxidant vitamin E [90].

The activity of mitochondrial superoxide dismutase 2 (SOD2) is reduced in mice exposed to CS. Notably, Apoe^−/−^Sod2^+/−^ mice displayed significant increases in atherosclerotic lesion formation compared with control Apoe^−/−^ littermates [91]. Thus, increased mitochondrial oxidant production, which could be influenced by mitochondrial and nuclear genetic variation in humans, may directly enhance atherogenic susceptibility.

Apoe^−/−^ mouse models are also appropriate to investigate the atherosclerotic effects of other pollutants such as combustion emissions and ambient air fine particulate matter [92–94], which induce inflammatory responses and generate reactive oxygen species. Thus, as previously described [94], Apoe^−/−^ mice have been used in a diverse range of inhalation toxicology studies. These studies have assessed exposure to motor vehicle emissions, environmental air particles, and mainstream and sidestream CS, with or without the effects of a high-fat diet. The delivery methods and exposure times varied between studies. The pathophysiological events, including inflammation, thrombosis, CVD, oxidative stress, and atherogenesis (Table 1), and endpoints such as plaque development, protein marker expression, and systems toxicology assessments, also varied among the studies.

## Apoe^−/−^ mouse model to investigate toxicological mechanisms of CS, smoking cessation, and harm reduction approaches

CS is a complex aerosol mixture, for which more than 8000 identified smoke constituents have been reported [95]. Although it is not clear which particular CS constituents plays which role in any specific disease pathways, there is a general consensus on the main classes of chemicals from the combustion of tobacco that are harmful and potentially harmful [35, 96].

In the recent years, there has been a growing interest in harm reduction approaches to address the health risks of smoking [97–100]. Smoking cessation remains the most effective approach to minimizing the risk for smoking-related diseases [101–103]. However, only a small percentage of individuals manage to achieve long-term abstinence without support [104]. Although nicotine replacement therapies (NRT) (e.g., patches, gums, and nasal sprays) are often used as smoking cessation aids, they do not address the sensory and behavioral aspects of the smoking ritual, thus limiting their efficacy [105]. Therefore, there is a space for tobacco harm reduction approaches, which includes the development of less harmful products as alternatives to smoking cigarettes. These products are designed to deliver nicotine, sensory and behavioral aspects that smokers find satisfying while significantly reducing or eliminating the harmful and potentially harmful chemicals in CS and may be referred to as modified risk tobacco products (MRTPs). MRTPs were defined by the US Family Smoking Prevention and Tobacco Control Act of 2009 as “*any tobacco product that is sold or distributed for use to reduce harm or the risk of tobacco related disease associated with commercially marketed tobacco products*” [35]. Importantly, according with the US Food and Drug Administration published draft guidance on “modified risk tobacco product applications” [35], applications must provide scientific evidence to demonstrate that the product will “*significantly reduce harm and the risk of tobacco*-*related diseases to individual tobacco users, and benefit the health of the population as a whole, taking into account both users of tobacco products and persons who do not currently use tobacco products*.”

Combustion of tobacco results in both pyrolysis and pyrosynthesis of many harmful or potentially harmful constituents (HPHCs) [106–108]. As the health risk associated with CS is due to the HPHCs it contains, the main driver for risk reduction is the reduction in exposure to HPHCs. Therefore, preventing combustion can produce, as compared with CS, a simpler aerosol with a strongly reduced content of these toxicants [19, 109, 110]. This principle underlies products based on the “heat-not-burn” principle, in which tobacco is electrically heated in a controlled fashion to release nicotine and flavors.

In this context, non-clinical studies in Apoe^−/−^ mice are an invaluable preclinical tool for the evaluation of candidate MRTPs (cMRTPs), as the effect of such products can be compared with well characterized CS effects in the Apoe^−/−^ mouse model (see Table 2).

Using a systems toxicology approach [111, 112], we were able to show that CS activates several response pathways in both respiratory (lung and nasal) and non-respiratory (liver, heart, and aorta) tissues. These responses, including inflammation, cell proliferation, lipid accumulation, complement, and tissue remodeling, were deactivated or strongly attenuated in Apoe^−/−^ mice upon smoking cessation, as well as upon the exposure to aerosol from a cMRTP [18, 19, 113–115].

Among these studies, particular interest was brought to the investigation of lipidomic profiles in different tissues (plasma, aorta, liver, and lungs) of Apoe^−/−^ mice exposed to CS. CS increased the levels of multiple lipids, including ceramides, cholesteryl esters, and phosphatidylcholine species, and increased the development of atherosclerotic plaques [27, 103]. One study conducted in 2012 confirmed these observations [18, 116, 117]. The study results showed the effects exerted by both CS (3-month exposure) and cessation (3-month CS + 3 months fresh air) on Apoe^−/−^ mice plasma and vascular tissue lipidomic profile. The CS-dependent increase of plasma and aortic lipid levels was reversed following smoking cessation, with a consequent decrease of most lipid concentrations, including total cholesterol, VLDL, phosphatidylcholine, and sphingomyelin [116, 117]. More recently, by using a systems toxicology approach, exposure effects were investigated using the classical toxicological endpoints related to both atherosclerosis and respiratory diseases, such as physiology and histology, combined with in depth molecular characterization of the transcriptome, proteome and lipidome [19, 94].

This study, together with others developed as part of a more comprehensive systems toxicology assessment framework [112–115], confirmed previous observations showing a CS-dependent increase of atherogenic lipid composition of plasma and vascular tissue. Phillips and colleagues used a different timing for the analysis, exposing mice up to 8 months [19]. The cessation group was defined as the group receiving 2 months of CS and 6 months of fresh air. The recovery in the cessation group in this particular condition was much more pronounced than in the previous study with a 3-month CS exposure plus 3-month cessation period [117]. This is consistent with the assumption that shorter exposure periods combined with longer post-exposure periods are more efficient at restoring lipids back to less atherogenic levels.

**Table 2:** Summary of studies using Apoe^−/−^ mice conducted by PMI

Disease, mechanism	References	Biological matrix	Endpoint	Effect of CS exposure	Effect of cessation
CVD	[116]	Aorta	Lipidomics	↑↗	ND
CVD	[117]	Aorta	Lipidomics	↑	↓→
CVD	[19]	Aorta	Lipidomics	↑	↓→
CVD	[42]	Carotid artery	Thrombosis	↑	ND
CVD	[42]	Carotid artery	Endothelial injury	↑	ND
CVD	[116]	Liver	Lipidomics	↑→	ND
CVD	[118]	Liver	Lipidomics	↑↗	↓↑
CVD	[114]	Liver	Lipidomics	↑↗	↓↘
CVD	[118]	Liver	Transcriptomics	→↗	→
CVD	[114]	Liver	Transcriptomics	↑↗	↓↘
CVD	[114]	Liver	Proteomics	↑↗	↓↘
CVD	[116]	Plasma	Lipidomics	↑↗	ND
CVD	[117]	Plasma	Lipidomics	↑→	↓→
CVD	[19]	Plasma	Lipidomics	↑↗	↓↘
COPD	[113]	Lung	Lipidomics	↑↗	↓↘
COPD	[113]	Lung	Transcriptomics	↑↗	↓↘
COPD	[113]	Lung	Proteomics	↑↗	↓↘
Atherosclerosis	[116]	Aorta	Plaque size	↑	ND
Atherosclerosis	[117]	Aorta	Plaque size	↑↘	↘
Atherosclerosis	[19]	Aorta	Plaque size	↑	→
Atherosclerosis	[118]	Aorta	Plaque size	↑	ND
Atherosclerosis	[29]	Aorta	Plaque size	↑	ND
Atherosclerosis	[27]	BA	Plaque size	↑→	ND
Atherosclerosis	[27]	Aortic arch	Plaque size	↑→	ND
Exposure markers	[18]	Blood	COHb	↑	↓
Exposure markers	[18]	Urine	Nicotine metabolites	↑	↓
Exposure marker	[117]	Blood	COHb	↑	↓
Exposure marker	[118]	Blood	COHb	↑	↓
Exposure marker	[117]	Urine	Nicotine metabolites	↑	↓
Exposure markers	[19]	Blood	COHb	↑	↓
Exposure markers	[19]	Urine	Nicotine metabolites	↑	↓
Inflammation	[18]	Lung	BALF	↑	↓↘
Inflammation	[18]	NRE	Histopathology	↑	↓
Inflammation	[18]	Lung	Transcriptomics	↑↓	↓↑
Inflammation	[116]	Liver	Transcriptomics	↑↗	ND
Inflammation	[124]	Lung	BALF	↑→	↓
Inflammation	[29]	Aorta	Transcriptomics	↑	ND
Inflammation	[127]	Lung	Transcriptomics	↑↓	ND
Inflammation	[127]	Lung	BALF	↑	ND
Inflammation	[127]	Respiratory	Histopathology	↑	ND
Inflammation	[127]	Respiratory	Network model	↑	ND
Inflammation	[113]	Lung	Lipidomics	↑	↓
Inflammation	[19]	Urine	Inflammatory biomarker	↑	↓
Inflammation	[19]	Lung	BALF	↑	↓
Inflammation	[19]	NRE	Histopathology	↑	↓
Inflammation	[19]	Lung	Histopathology	↑	↓
COPD—emphysema	[18]	Lung	Lung morphometry	↑	↘
COPD—emphysema	[19]	Lung	Lung function	↑	↘
COPD—emphysema	[19]	Lung	Lung morphometry	↑	↘
Inflammation/cell cycle/necroptosis	[19]	Lung	Network model	↑	↓
Inflammation/cell cycle/necroptosis	[19]	Lung	Transcriptomics	↑	↓
Inflammation/cell cycle/necroptosis	[19]	Lung	Proteomics	↑	↓
Inflammation/cell cycle/necroptosis	[124]	Lung	Network model	↑	↓
Inflammation/cell cycle/necroptosis	[124]	Lung	Transcriptomics	↑	↓
Oxidative stress	[18]	Lung	BALF	↑	↓↘
Oxidative stress	[18]	NRE	Transcriptomics	↑↓	↓↑
Oxidative stress	[114]	Liver	Transcriptomics	↑	↓
Oxidative stress	[114]	Liver	Proteomics	↑	↓

Arrows indicate increase (upwards), decrease (downwards), no change (to the right), trend to increase (north east), and trend to decrease (south east). Arrows for the cigarette smoke (CS) effect indicate changes compared with unexposed mice, and arrows for the cessation effect indicate differences compared with continued CS exposure

*COHb* carboxyhemoglobin; *BALF* bronchoalveolar lavage fluid; *CVD* cardiovascular disease; *NRE* nasal respiratory epithelium; *COPD* chronic obstructive pulmonary disease; *BA* brachiocephalic artery; *ND* not done

The effects of CS exposure and smoking cessation were also determined on hepatic lipid, transcriptomic, and proteomic profiles in Apoe^−/−^ mice [114, 116, 118]. De Leon and colleagues [118] showed that the concentrations of many lipids, including free cholesterol, ceramides, and sphingomyelin, decreased in the liver of Apoe^−/−^ mice following smoking cessation, although the triacylglycerol concentration increased. Hepatic triacylglycerol accumulation is indicative of fatty liver disease, but the observed increase did not appear to reflect decreased hepatic lipolysis or decreased pancreatic lipase. Gene set enrichment analysis of the transcriptomes revealed that fewer genes were differentially regulated in the smoking cessation group than in the CS exposed group. Genes showing differential expression included those involved in the cell cycle, cholesterol biosynthesis, and platelet activation. A more recent study partially confirmed these results [114]. In fact, analysis of the liver proteome and transcriptome showed upregulation of several enzymes involved in fatty acid and cholesterol metabolism upon CS inhalation. It should also be noted that the Apoe deficiency *per se* already causes a skewed hepatic lipid metabolism and a higher baseline of hepatic triglyceride accumulation compared with wild-type mice [119, 120]. Therefore, while signs of overt hepatotoxicity were absent, livers of Apoe^−/−^ mice exposed to CS did exhibit an exacerbated dysregulation of fundamental hepatic processes such as lipid, xenobiotic and iron homeostasis compared with the sham (fresh air exposed) group. These data suggested that smoking alone cannot be regarded as a causative agent for liver diseases but, rather, as an accelerator in the presence of synergistic factors (alcohol, chronic liver diseases) [121–123]. Finally, these changes were significantly reduced or absent when mice were exposed continuously to the cMRTP aerosol, upon cessation regimen or switching to the cMRTP [114].

Thomson and colleagues [124] developed a means of assessing the biological impact of exposure to biological and chemical substances based on hypotheses (typically, the predicted upstream regulators of the measured differentially expressed genes), subnetworks, and networks. Transcriptomics data were evaluated by computational modeling that combined the data with a priori knowledge from a knowledgebase to determine the effects at the molecular and systems levels. This approach has been applied to a number of models, including two smoking cessation studies using Apoe^−/−^ mice [18, 19]. Both these studies revealed that in respiratory tract tissues, genes involved in the cell cycle, pulmonary inflammation, senescence, and necroptosis were significantly perturbed by CS exposure. This was corroborated by a decrease in the perturbation of biological mechanisms related to cell proliferation, tissue repair/angiogenesis, cell stress, cell fate, and pulmonary inflammation in the smoking cessation group compared with the group under continuous CS exposure. Similarly, the predicted increase in the immune cell response subnetworks was supported by a significant increase in the immune cell count and their secreted inflammatory mediators in bronchoalveolar lavage fluid.

Because advanced atherosclerotic lesions are difficult to recreate experimentally, little is known about the mechanisms underlying plaque instability and rupture. Instead, this was modeled in a vascular inflammatory processes network [29], which, like the lung-specific models applied for respiratory tract evaluations, used reverse causal reasoning [125] to explain and integrate differential gene expression data from large datasets. The latter approach showed that distinct molecular pathways are involved in different stages of atherosclerosis in Apoe^−/−^ mice exposed to CS. Thus, the Apoe^−/−^ mouse model shares many causal mechanisms with those of advanced atherosclerotic lesions in human coronary arteries, including endothelial cell activation, endothelial/monocyte interaction, foam cell formation, and plaque destabilization.

**Fig. 4:**
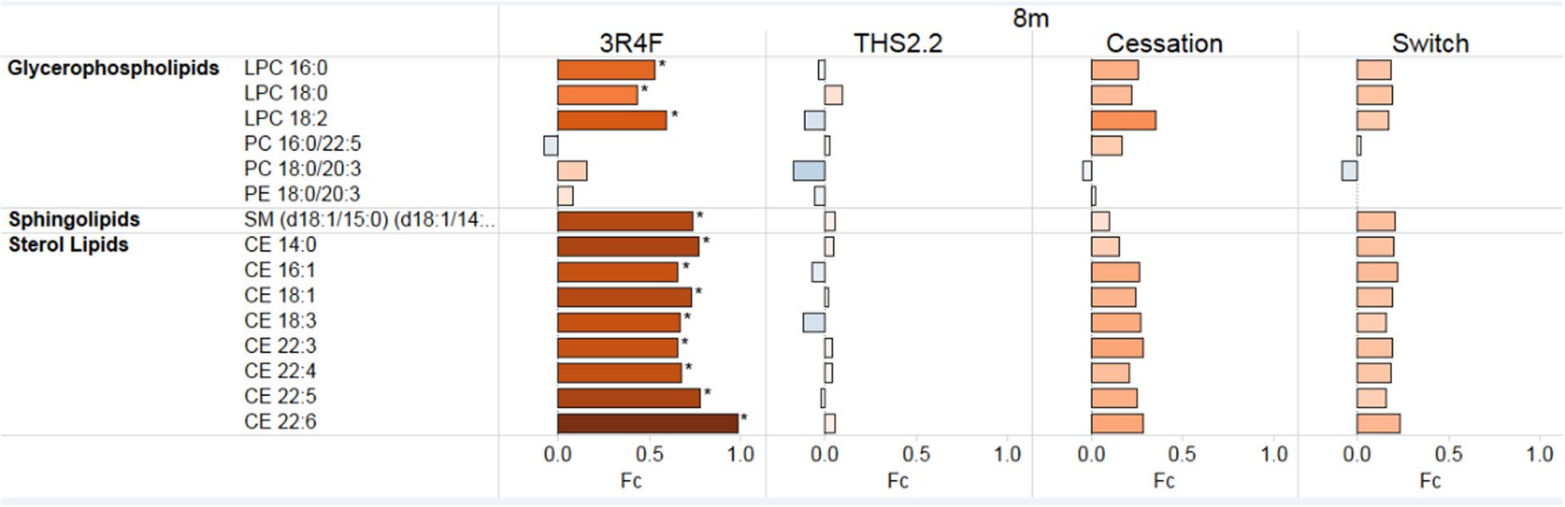
Aortic arch lipids from exposed Apoe^−/−^ mice vs sham controls shared with the human plaque-enriched lipids reported by Stegemann et al. [126]. Differential abundance profiles for lipid species with significant differential abundance in any exposure to sham comparison (*BH-adjusted *p* value < 0.05). The *x-axis* and the *color code* indicate the log_2_ (Fc)

Stegemann and colleagues analyzed the lipid composition of human atherosclerotic plaques from carotid endarterectomies and reported a list of plaque-enriched lipid species, also with a strong contribution from sterol lipids, sphingolipids, and glycerophospholipids [126]. In Fig. 4, we present a comparison of the Apoe^−/−^ murine aortic arch lipid species (data from [19]) that have these human plaque-enriched lipids in common. With the exception of two phosphatidylcholines (PCs) and one phosphatidylethanolamine (PE), all these common lipids were also significantly higher in aortic arches from Apoe^−/−^ mice exposed for 8 months to CS compared with tissue from the sham-exposed controls. Conversely, no significant changes were observed following exposure to aerosol from an electrically heated cMRTP, or in mice that were switched to the cMRTP or to fresh air (cessation group) after a 2-month CS exposure. This correlation between human and murine plaque-enriched lipids is suggestive of related alterations in lipid metabolism in this mouse model of atherosclerosis. Taken together, these findings suggest that similar mechanisms are common to both species at the early stages of smoking-related atherosclerosis.

Another study used a systems biology approach together with reverse causal reasoning to investigate the mechanisms associated with emphysema development in five different mouse models, including Apoe^−/−^ mice [127]. Emphysema is a component of COPD, but it appears to have a strong underlying genetic element in its development because not all smokers develop this disease. Combining large-scale transcriptomics data sets from four different studies, 39 biological and chemical substances based on hypotheses were identified as potential mechanisms associated with emphysema induced by CS exposure. The transcription nuclear factor κB, Th1-associated cytokines (tumor necrosis factor-α, IL-2, and interferon-c), IL-17A and IL-17F were predicted to increase following CS exposure. These fundamental mechanisms are likely to be applicable to human COPD.

## Conclusions

To understand disease mechanisms in human, it is sometimes necessary to use animal models that can be characterized in depth. Oftentimes, models are used to study one particular condition. It is possible, however, to bring the model closer to the human situation by studying multiple comorbidities at once, as it is discussed in this review for the Apoe^−/−^ mouse. Although atherogenesis is accelerated in Apoe^−/−^ mice compared with humans, this rodent model is useful for assessing the mechanisms underlying the progression of atherosclerosis that are also relevant to humans owing to the similarities in the underlying mechanisms. In this review article we discuss scientific publications that overall demonstrated that the Apoe^−/−^ mouse model is a suitable and relevant tool for examining the effects of CS, smoking cessation, or any product aimed at reducing the risk associated with CS, not only on atherosclerosis but also on lung inflammation and emphysema. In this context, the versatility of the Apoe^−/−^ mouse model is highlighted by the possibility not only to allow analysis in terms of genetic, molecular, and physiological mechanisms in cardiovascular systems, but also to apply a similar “systems” approach to other respiratory and non-respiratory tissues. Thus, the use of the Apoe^−/−^ mouse model appears very appropriate in the framework of inhalation toxicology studies.

## References

[1] Jin ZX, Xiong Q, Jia F, Sun CL, Zhu HT, Ke FS (2015). Investigation of RNA interference suppression of matrix metalloproteinase-9 in mouse model of atherosclerosis. Int J Clin Exp Med.

[2] Yahagi K, Davis HR, Arbustini E, Virmani R (2015). Sex differences in coronary artery disease: pathological observations. Atherosclerosis.

[3] Libby P, Pasterkamp G (2015). Requiem for the ‘vulnerable plaque’. Eur Heart J.

[4] Bairey Merz CN, Mark S, Boyan BD, Jacobs AK, Shah PK, Shaw LJ, Taylor D, Marbán E (2010). Proceedings from the scientific symposium: sex differences in cardiovascular disease and implications for therapies. J Womens Health (Larchmt).

[5] Mozaffarian D, Benjamin EJ, Go AS, Arnett DK, Blaha MJ, Cushman M, de Ferranti S, Despres JP, Fullerton HJ, Howard VJ (2015). Heart disease and stroke statistics–2015 update: a report from the American Heart Association. Circulation.

[6] Zaragoza C, Gomez-Guerrero C, Martin-Ventura JL, Blanco-Colio L, Lavin B, Mallavia B, Tarin C, Mas S, Ortiz A, Egido J (2011). Animal models of cardiovascular diseases. J Biomed Biotechnol.

[7] Véniant MM, Withycombe S, Young SG (2001). Lipoprotein size and atherosclerosis susceptibility in Apoe^−/−^ and Ldlr^−/−^ mice. Arterioscler Thromb Vasc Biol.

[8] Ishibashi S, Brown MS, Goldstein JL, Gerard RD, Hammer RE, Herz J (1993). Hypercholesterolemia in low density lipoprotein receptor knockout mice and its reversal by adenovirus-mediated gene delivery. J Clin Invest.

[9] Zhang SH, Reddick RL, Piedrahita JA, Maeda N (1992). Spontaneous hypercholesterolemia and arterial lesions in mice lacking apolipoprotein E. Science.

[10] Nakashima Y, Plump AS, Raines EW, Breslow JL, Ross R (1994). ApoE-deficient mice develop lesions of all phases of atherosclerosis throughout the arterial tree. Arterioscler Thromb Vasc Biol.

[11] Shaw PX (2004). Rethinking oxidized low-density lipoprotein, its role in atherogenesis and the immune responses associated with it. Arch Immunol Ther Exp (Warsz).

[12] Bink DI, Ritz K, Aronica E, van der Weerd L, Daemen MJ (2013). Mouse models to study the effect of cardiovascular risk factors on brain structure and cognition. J Cereb Blood Flow Metab.

[13] Choi J, Forster MJ, McDonald SR, Weintraub ST, Carroll CA, Gracy RW (2004). Proteomic identification of specific oxidized proteins in ApoE-knockout mice: relevance to Alzheimer’s disease. Free Radic Biol Med.

[14] Crisby M, Rahman S, Sylven C, Winblad B, Schultzberg M (2004). Effects of high cholesterol diet on gliosis in apolipoprotein E knockout mice: implications for Alzheimer’s disease and stroke. Neurosci Lett.

[15] Behr-Roussel D, Darblade B, Oudot A, Compagnie S, Bernabé J, Alexandre L, Giuliano F (2006). Erectile dysfunction in hypercholesterolemic atherosclerotic apolipoprotein E knockout mice. J Sex Med.

[16] Tous M, Ferré N, Camps J, Riu F, Joven J (2005). Feeding apolipoprotein E-knockout mice with cholesterol and fat enriched diets may be a model of non-alcoholic steatohepatitis. Mol Cell Biochem.

[17] Bonomini F, Rodella L, Moghadasian M, Lonati C, Rezzani R (2013). Apolipoprotein E deficiency and a mouse model of accelerated liver aging. Biogerontology.

[18] Boué S, De León H, Schlage WK, Peck MJ, Weiler H, Berges A, Vuillaume G, Martin F, Friedrichs B, Lebrun S (2013). Cigarette smoke induces molecular responses in respiratory tissues of ApoE^−/−^ mice that are progressively deactivated upon cessation. Toxicology.

[19] Phillips B, Veljkovic E, Boue S, Schlage WK, Vuillaume G, Martin F, Titz B, Leroy P, Buettner A, Elamin A (2016). An 8-month systems toxicology inhalation/cessation study in Apoe^−/−^ mice to investigate cardiovascular and respiratory exposure effects of a candidate modified risk tobacco product, THS 2.2, compared with conventional cigarettes. Toxicol Sci.

[20] Goldklang M, Golovatch P, Zelonina T, Trischler J, Rabinowitz D, Lemaître V, D’Armiento J (2012). Activation of the TLR4 signaling pathway and abnormal cholesterol efflux lead to emphysema in ApoE-deficient mice. Am J Physiol Lung Cell Mol Physiol.

[21] Han SG, Howatt DA, Daugherty A, Gairola CG (2012). Atherogenic and pulmonary responses of ApoE-and LDL receptor-deficient mice to sidestream cigarette smoke. Toxicology.

[22] Khedoe PP, Wong MC, Wagenaar GT, Plomp JJ, van Eck M, Havekes LM, Rensen PC, Hiemstra PS, Berbee JF (2013). The effect of PPE-induced emphysema and chronic LPS-induced pulmonary inflammation on atherosclerosis development in APOE*3-LEIDEN mice. PLoS One.

[23] Hoekstra M, Van Eck M (2015). Mouse models of disturbed HDL metabolism. Handb Exp Pharmacol.

[24] Zanoni P, Khetarpal SA, Larach DB, Hancock-Cerutti WF, Millar JS, Cuchel M, DerOhannessian S, Kontush A, Surendran P, Saleheen D (2016). Rare variant in scavenger receptor BI raises HDL cholesterol and increases risk of coronary heart disease. Science.

[25] Fuller M, Dadoo O, Serkis V, Abutouk D, MacDonald M, Dhingani N, Macri J, Igdoura SA, Trigatti BL (2014). The effects of diet on occlusive coronary artery atherosclerosis and myocardial infarction in scavenger receptor class B, type 1/low-density lipoprotein receptor double knockout mice. Arterioscler Thromb Vasc Biol.

[26] Jawien J (2012). The role of an experimental model of atherosclerosis: apoE-knockout mice in developing new drugs against atherogenesis. Curr Pharm Biotechnol.

[27] Von Holt K, Lebrun S, Stinn W, Conroy L, Wallerath T, Schleef R (2009). Progression of atherosclerosis in the Apo E^−/−^ model: 12-Month exposure to cigarette mainstream smoke combined with high-cholesterol/fat diet. Atherosclerosis.

[28] Tamminen M, Mottino G, Qiao J, Breslow J, Frank J (1999). Ultrastructure of early lipid accumulation in ApoE-deficient mice. Arterioscler Thromb Vasc Biol.

[29] De Leon H, Boue S, Schlage WK, Boukharov N, Westra JW, Gebel S, VanHooser A, Talikka M, Fields RB, Veljkovic E (2014). A vascular biology network model focused on inflammatory processes to investigate atherogenesis and plaque instability. J Transl Med.

[30] Tse J, Martin-McNaulty B, Halks-Miller M, Kauser K, DelVecchio V, Vergona R, Sullivan ME, Rubanyi GM (1999). Accelerated atherosclerosis and premature calcified cartilaginous metaplasia in the aorta of diabetic male Apo E knockout mice can be prevented by chronic treatment with 17β-estradiol. Atherosclerosis.

[31] Gräbner R, Lötzer K, Döpping S, Hildner M, Radke D, Beer M, Spanbroek R, Lippert B, Reardon CA, Getz GS (2009). Lymphotoxin β receptor signaling promotes tertiary lymphoid organogenesis in the aorta adventitia of aged ApoE^−/−^ mice. J Exp Med.

[32] Havel RJ, Kane, JP. Introduction: structure and metabolism of plasma lipoprotein. In: Scriver CR, Beaudet AL, Sly WS, Valle D, editors. The metabolic basis of inherited disease. New York: McGraw-Hill; 1989. p. 1129–38.

[33] Smith J, Breslow J (1997). The emergence of mouse models of atherosclerosis and their relevance to clinical research. J Intern Med.

[34] Daugherty A, Rateri DL (2005). Development of experimental designs for atherosclerosis studies in mice. Methods.

[35] Food and drug administration F. Modified risk tobacco product applications: draft guidance for industry. 2012.

[36] Nussbaumer-Ochsner Y, Rabe KF (2011). Systemic manifestations of COPD. Chest.

[37] Hansell AL, Walk JA, Soriano JB (2003). What do chronic obstructive pulmonary disease patients die from? A multiple cause coding analysis. Eur Respir J.

[38] Andrus MR, Loyed JV (2008). Use of beta-adrenoceptor antagonists in older patients with chronic obstructive pulmonary disease and cardiovascular co-morbidity: safety issues. Drugs Aging.

[39] Kodavanti UP, Costa DL (2001). Rodent models of susceptibility: what is their place in inhalation toxicology?. Respir Physiol.

[40] Massaro D, Massaro GD (2008). Apoetm1Unc mice have impaired alveologenesis, low lung function, and rapid loss of lung function. Am J Physiol Lung Cell Mol Physiol.

[41] Arunachalam G, Sundar IK, Hwang JW, Yao H, Rahman I (2010). Emphysema is associated with increased inflammation in lungs of atherosclerosis-prone mice by cigarette smoke: implications in comorbidities of COPD. J Inflamm.

[42] Schroeter MR, Sawalich M, Humboldt T, Leifheit M, Meurrens K, Berges A, Xu H, Lebrun S, Wallerath T, Konstantinides S (2008). Cigarette smoke exposure promotes arterial thrombosis and vessel remodeling after vascular injury in apolipoprotein E-deficient mice. J Vasc Res.

[43] Knight-Lozano CA, Young CG, Burow DL, Hu ZY, Uyeminami D, Pinkerton KE, Ischiropoulos H, Ballinger SW (2002). Cigarette smoke exposure and hypercholesterolemia increase mitochondrial damage in cardiovascular tissues. Circulation.

[44] Biswas SK, Newby DE, Rahman I, Megson IL (2005). Depressed glutathione synthesis precedes oxidative stress and atherogenesis in Apo-E^−/−^ mice. Biochem Biophys Res Commun.

[45] Schick S, Glantz S (2005). Philip Morris toxicological experiments with fresh sidestream smoke: more toxic than mainstream smoke. Tob Control.

[46] Leavitt BJ, Ross CS, Spence B, Surgenor SD, Olmstead EM, Clough RA, Charlesworth DC, Kramer RS, O’Connor GT, Group NNECDS (2006). Long-term survival of patients with chronic obstructive pulmonary disease undergoing coronary artery bypass surgery. Circulation.

[47] Wilson PW, Schaefer EJ, Larson MG, Ordovas JM (1996). Apolipoprotein E alleles and risk of coronary disease A meta-analysis. Arterioscler Thromb Vasc Biol.

[48] Davignon J, Gregg RE, Sing CF (1988). Apolipoprotein E polymorphism and atherosclerosis. Arterioscler Thromb Vasc Biol.

[49] Knouff C, Hinsdale ME, Mezdour H, Altenburg MK, Watanabe M, Quarfordt SH, Sullivan PM, Maeda N (1999). Apo E structure determines VLDL clearance and atherosclerosis risk in mice. J Clin Invest.

[50] Windler E, Kovanen PT, Chao Y-S, Brown M, Havel RJ, Goldstein J (1980). The estradiol-stimulated lipoprotein receptor of rat liver. A binding site that membrane mediates the uptake of rat lipoproteins containing apoproteins B and E. J Biol Chem.

[51] Brown MS, Goldstein JL (1986). A receptor-mediated pathway for cholesterol homeostasis. Science.

[52] Mahley RW (1988). Apolipoprotein E: cholesterol transport protein with expanding role in cell biology. Science.

[53] Rea IM, Mc Dowell I, McMaster D, Smye M, Stout R, Evans A (2001). Apolipoprotein E alleles in nonagenarian subjects in the belfast elderly longitudinal free-living ageing study (BELFAST). Mech Ageing Dev.

[54] Kolovou G, Yiannakouris N, Hatzivassiliou M, Malakos J, Daskalova D, Hatzigeorgiou G, Cariolou MA, Cokkinos DV (2002). Association of apolipoprotein E polymorphism with myocardial infarction in Greek patients with coronary artery disease. Curr Med Res Opin.

[55] Kolovou GD, Anagnostopoulou KK, Mikhailidis DP, Panagiotakos DB, Pilatis ND, Cariolou MA, Yiannakouris N, Degiannis D, Stavridis G, Cokkinos DV (2005). Association of apolipoprotein E genotype with early onset of coronary heart disease in Greek men. Angiology.

[56] Corder E, Saunders A, Strittmatter W, Schmechel D, Gaskell P, Small G, Roses A, Haines J, Pericak-Vance MA (1993). Gene dose of apolipoprotein E type 4 allele and the risk of Alzheimer’s disease in late onset families. Science.

[57] Bretsky P, Guralnik J, Launer L, Albert M, Seeman TE (2003). The role of APOE-ε4 in longitudinal cognitive decline MacArthur studies of successful aging. Neurology.

[58] Kolovou GD, Anagnostopoulou KK, Cokkinos DV (1022). Apolipoprotein epsilon4 homozygosity and essential hypertension. South Med J.

[59] Meade T, Brozovic M, Chakrabarti R, Haines A, Imeson J, Mellows S, Miller G, North W, Stirling Y, Thompson S (1986). Haemostatic function and ischaemic heart disease: principal results of the Northwick Park heart study. Lancet.

[60] Humphries SE, Talmud PJ, Hawe E, Bolla M, Day IN, Miller GJ (2001). Apolipoprotein E4 and coronary heart disease in middle-aged men who smoke: a prospective study. Lancet.

[61] Talmud P, Stephens J, Hawe E, Demissie S, Cupples L, Hurel S, Humphries S, Ordovas J (2005). The significant increase in cardiovascular disease risk in APOEɛ4 carriers is evident only in men who smoke: potential relationship between reduced antioxidant status and APOE4. Ann Hum Genet.

[62] Flecknell P (2002). Replacement, reduction and refinement. ALTEX.

[63] Russell WMS, Burch RL, Hume CW. The principles of humane experimental technique. 1959.

[64] Rothblat GH, Mahlberg FH, Johnson W, Phillips MC (1992). Apolipoproteins, membrane cholesterol domains, and the regulation of cholesterol efflux. J Lipid Res.

[65] Andrikoula M, McDowell I (2008). The contribution of ApoB and ApoA1 measurements to cardiovascular risk assessment. Diabetes Obes Metab.

[66] Borén J, Lee I, Zhu W, Arnold K, Taylor S, Innerarity TL (1084). Identification of the low density lipoprotein receptor-binding site in apolipoprotein B100 and the modulation of its binding activity by the carboxyl terminus in familial defective apo-B100. J Clin Invest.

[67] Hussain MM (2014). Intestinal lipid absorption and lipoprotein formation. Curr Opin Lipidol.

[68] Goldstein JL, Kita T, Brown MS (1983). Defective lipoprotein receptors and atherosclerosis. Lessons from an animal counterpart of familial hypercholesterolemia. N Engl J Med.

[69] Williams KJ (2008). Molecular processes that handle—and mishandle—dietary lipids. J Clin Invest.

[70] Willnow TE (1997). Mechanisms of hepatic chylomicron remnant clearance. Diabet Med.

[71] Lillis AP, Van Duyn LB, Murphy-Ullrich JE, Strickland DK (2008). LDL receptor-related protein 1: unique tissue-specific functions revealed by selective gene knockout studies. Physiol Rev.

[72] Shiomi M, Ishida T, Koike T (2012). Genetically modified animal models for lipoprotein research.

[73] Olofsson SO, Borén J (2012). Apolipoprotein B secretory regulation by degradation. Arterioscler Thromb Vasc Biol.

[74] Greeve J, Altkemper I, Dieterich JH, Greten H, Windler E (1993). Apolipoprotein B mRNA editing in 12 different mammalian species: hepatic expression is reflected in low concentrations of apoB-containing plasma lipoproteins. J Lipid Res.

[75] Takahashi S, Sakai J, Fujino T, Hattori H, Zenimaru Y, Suzuki J, Miyamori I, Yamamoto TT (2004). The very low-density lipoprotein (VLDL) receptor: characterization and functions as a peripheral lipoprotein receptor. J Atheroscler Thromb.

[76] Li X, Catalina F, Grundy S, Patel S (1996). Method to measure apolipoprotein B-48 and B-100 secretion rates in an individual mouse: evidence for a very rapid turnover of VLDL and preferential removal of B-48-relative to B-100-containing lipoproteins. J Lipid Res.

[77] Agellon L, Walsh A, Hayek T, Moulin P, Jiang XC, Shelanski SA, Breslow J, Tall AR (1991). Reduced high density lipoprotein cholesterol in human cholesteryl ester transfer protein transgenic mice. J Biol Chem.

[78] Rohrer L, Ohnsorg PM, Lehner M, Landolt F, Rinninger F, von Eckardstein A (2009). High-density lipoprotein transport through aortic endothelial cells involves scavenger receptor BI and ATP-binding cassette transporter G1. Circ Res.

[79] Son Y, Zilversmit D (1986). Increased lipid transfer activities in hyperlipidemic rabbit plasma. Arterioscler Thromb Vasc Biol.

[80] Gairola CG, Drawdy ML, Block AE, Daugherty A (2001). Sidestream cigarette smoke accelerates atherogenesis in apolipoprotein E^−/−^ mice. Atherosclerosis.

[81] Gairola CG, Howatt DA, Daugherty A (2010). Dietary coenzyme Q10 does not protect against cigarette smoke-augmented atherosclerosis in apoE-deficient mice. Free Radic Biol Med.

[82] Takagi H, Umemoto T (2005). Smoking promotes pathogenesis of aortic aneurysm through the 5-lipoxygenase pathway. Med Hypotheses.

[83] Stolle K, Berges A, Lietz M, Lebrun S, Wallerath T (2010). Cigarette smoke enhances abdominal aortic aneurysm formation in angiotensin II-treated apolipoprotein E-deficient mice. Toxicol Lett.

[84] Matetzky S, Tani S, Kangavari S, Dimayuga P, Yano J, Xu H, Chyu KY, Fishbein MC, Shah PK, Cercek B (2000). Smoking increases tissue factor expression in atherosclerotic plaques: implications for plaque thrombogenicity. Circulation.

[85] Dong A, Caicedo J, Han SG, Mueller P, Saha S, Smyth SS, Gairola CG (2010). Enhanced platelet reactivity and thrombosis in Apoe^−/−^ mice exposed to cigarette smoke is attenuated by P2Y12 antagonism. Thromb Res.

[86] Bliden KP, Dichiara J, Lawal L, Singla A, Antonino MJ, Baker BA, Bailey WL, Tantry US, Gurbel PA (2008). The association of cigarette smoking with enhanced platelet inhibition by clopidogrel. J Am Coll Cardiol.

[87] Barbieri SS, Weksler BB (2007). Tobacco smoke cooperates with interleukin-1beta to alter beta-catenin trafficking in vascular endothelium resulting in increased permeability and induction of cyclooxygenase-2 expression in vitro and in vivo. FASEB J.

[88] Barbieri SS, Ruggiero L, Tremoli E, Weksler BB (2008). Suppressing PTEN activity by tobacco smoke plus interleukin-1beta modulates dissociation of VE-cadherin/beta-catenin complexes in endothelium. Arterioscler Thromb Vasc Biol.

[89] Tani S, Dimayuga PC, Anazawa T, Chyu KY, Li H, Shah PK, Cercek B (2004). Aberrant antibody responses to oxidized LDL and increased intimal thickening in apoE^−/−^ mice exposed to cigarette smoke. Atherosclerosis.

[90] Kunitomo M, Yamaguchi Y, Kagota S, Yoshikawa N, Nakamura K, Shinozuka K (2009). Biochemical evidence of atherosclerosis progression mediated by increased oxidative stress in apolipoprotein E-deficient spontaneously hyperlipidemic mice exposed to chronic cigarette smoke. J Pharmacol Sci.

[91] Harrison CM, Pompilius M, Pinkerton KE, Ballinger SW (2011). Mitochondrial oxidative stress significantly influences atherogenic risk and cytokine-induced oxidant production. Environ Health Perspect.

[92] Seilkop SK, Campen MJ, Lund AK, McDonald JD, Mauderly JL (2012). Identification of chemical components of combustion emissions that affect pro-atherosclerotic vascular responses in mice. Inhal Toxicol.

[93] Chen LC, Quan C, Hwang JS, Jin X, Li Q, Zhong M, Rajagopalan S, Sun Q (2010). Atherosclerosis lesion progression during inhalation exposure to environmental tobacco smoke: a comparison to concentrated ambient air fine particles exposure. Inhal Toxicol.

[94] De Leon H, Boue S, Szostak J, Peitsch MC, Hoeng J (2015). Systems biology research into cardiovascular disease: contributions of lipidomics-based approaches to biomarker discovery. Curr Drug Discov Technol.

[95] Rodgman A, Perfetti TA (2013). The chemical components of tobacco and tobacco smoke.

[96] Talhout R, Schulz T, Florek E, Van Benthem J, Wester P, Opperhuizen A (2011). Hazardous compounds in tobacco smoke. Int J Environ Res Public Health.

[97] Sweanor D, Alcabes P, Drucker E (2007). Tobacco harm reduction: how rational public policy could transform a pandemic. Int J Drug Policy.

[98] World Health Organization (2007). The scientific basis of tobacco product regulation.

[99] Surgeons RCoPa (2007). Harm reduction in nicotine addiction: helping people who can’t quit.

[100] Zeller M, Hatsukami D (2009). The strategic dialogue on tobacco harm reduction: a vision and blueprint for action in the US. Tob Control.

[101] Bakhru A, Erlinger TP (2005). Smoking cessation and cardiovascular disease risk factors: results from the third national health and nutrition examination survey. PLoS Med.

[102] Godtfredsen N, Lam T, Hansel T, Leon M, Gray N, Dresler C, Burns D, Prescott E, Vestbo J (2008). COPD-related morbidity and mortality after smoking cessation: status of the evidence. Eur Respir J.

[103] Gepner AD, Piper ME, Johnson HM, Fiore MC, Baker TB, Stein JH (2011). Effects of smoking and smoking cessation on lipids and lipoproteins: outcomes from a randomized clinical trial. Am Heart J.

[104] Hughes JR, Keely J, Naud S (2004). Shape of the relapse curve and long-term abstinence among untreated smokers. Addiction.

[105] McRobbie H, Bullen C, Hartmann-Boyce J, Hajek P (2014). Electronic cigarettes for smoking cessation and reduction. Cochrane Database Syst Rev.

[106] Hoffmann D, Rathkamp G (1972). Quantitative determination of fluorenes in cigarette smoke and their formation by pyrosynthesis. Anal Chem.

[107] Brunnemann KD, Djordjevic MV, Feng R, Hoffmann D (1990). Analysis and pyrolysis of some N-nitrosamino acids in tobacco and tobacco smoke. IARC Sci Publ.

[108] Hertz-Schunemann R, Ehlert S, Streibel T, Liu C, McAdam K, Baker RR, Zimmermann R (2015). High-resolution time and spatial imaging of tobacco and its pyrolysis products during a cigarette puff by microprobe sampling photoionisation mass spectrometry. Anal Bioanal Chem.

[109] Baker RR (2006). Smoke generation inside a burning cigarette: modifying combustion to develop cigarettes that may be less hazardous to health. Prog Energy Combust Sci.

[110] Borgerding MF, Bodnar JA, Chung HL, Mangan PP, Morrison CC, Risner CH, Rogers JC, Simmons DF, Uhrig MS, Wendelboe FN (1998). Chemical and biological studies of a new cigarette that primarily heats tobacco. Part 1. Chemical composition of mainstream smoke. Food Chem Toxicol.

[111] Hoeng J, Deehan R, Pratt D, Martin F, Sewer A, Thomson TM, Drubin DA, Waters CA, de Graaf D, Peitsch MC (2012). A network-based approach to quantifying the impact of biologically active substances. Drug Discov Today.

[112] Sturla SJ, Boobis AR, FitzGerald RE, Hoeng J, Kavlock RJ, Schirmer K, Whelan M, Wilks MF, Peitsch MC (2014). Systems toxicology: from basic research to risk assessment. Chem Res Toxicol.

[113] Titz B, Boue S, Phillips B, Talikka M, Vihervaara T, Schneider T, Nury C, Elamin A, Guedj E, Peck MJ (2016). Effects of cigarette smoke, cessation, and switching to two heat-not-burn tobacco products on lung lipid metabolism in C57BL/6 and Apoe^−/−^ mice-An integrative systems toxicology analysis. Toxicol Sci.

[114] Lo Sasso G, Titz B, Nury C, Boue S, Phillips B, Belcastro V, Schneider T, Dijon S, Baumer K, Peric D (2016). Effects of cigarette smoke, cessation and switching to a candidate modified risk tobacco product on the liver in Apoe mice—a systems toxicology analysis. Inhal Toxicol.

[115] Szostak J, Boué S, Talikka M, Guedj E, Martin F, Phillips B, Ivanov N, Peitsch MC, Hoeng J. Long term exposure to cigarette smoke impacts the expression of genes involved in cardiac muscle structure and function in Apoe^−/−^ mouse while exposure to the aerosol of a tobacco heating system does not. BMC Med Genomics. 2015. (**personal communication**)

[116] Boué S, Tarasov K, Jänis M, Lebrun S, Hurme R, Schlage W, Lietz M, Vuillaume G, Ekroos K, Steffen Y (2012). Modulation of atherogenic lipidome by cigarette smoke in apolipoprotein E-deficient mice. Atherosclerosis.

[117] Lietz M, Berges A, Lebrun S, Meurrens K, Steffen Y, Stolle K, Schueller J, Boue S, Vuillaume G, Vanscheeuwijck P (2013). Cigarette-smoke-induced atherogenic lipid profiles in plasma and vascular tissue of apolipoprotein E-deficient mice are attenuated by smoking cessation. Atherosclerosis.

[118] De León H, Boué S, Peitsch M, Hoeng J (2013). Modulation of the hepatic lipidome and transcriptome of Apoe^−/−^mice in response to smoking cessation. J Liver.

[119] Wang L, Chen L, Tan Y, Wei J, Chang Y, Jin T, Zhu H (2013). Betaine supplement alleviates hepatic triglyceride accumulation of apolipoprotein E deficient mice via reducing methylation of peroxisomal proliferator-activated receptor alpha promoter. Lipids Health Dis.

[120] Mensenkamp AR, van Luyn MJ, Havinga R, Teusink B, Waterman IJ, Mann CJ, Elzinga BM, Verkade HJ, Zammit VA, Havekes LM (2004). The transport of triglycerides through the secretory pathway of hepatocytes is impaired in apolipoprotein E deficient mice. J Hepatol.

[121] Whitehead T, Robinson D, Allaway S (1996). The effects of cigarette smoking and alcohol consumption on serum liver enzyme activities: a dose-related study in men. Ann Clin Biochem.

[122] Azzalini L, Ferrer E, Ramalho LN, Moreno M, Domínguez M, Colmenero J, Peinado VI, Barbera JA, Arroyo V, Gines P (2010). Cigarette smoking exacerbates nonalcoholic fatty liver disease in obese rats. Hepatology.

[123] Altamirano J, Bataller R (2010). Cigarette smoking and chronic liver diseases. Gut.

[124] Thomson TM, Sewer A, Martin F, Belcastro V, Frushour BP, Gebel S, Park J, Schlage WK, Talikka M, Vasilyev DM (2013). Quantitative assessment of biological impact using transcriptomic data and mechanistic network models. Toxicol Appl Pharmacol.

[125] Catlett NL, Bargnesi AJ, Ungerer S, Seagaran T, Ladd W, Elliston KO, Pratt D (2013). Reverse causal reasoning: applying qualitative causal knowledge to the interpretation of high-throughput data. BMC Bioinformatics.

[126] Stegemann C, Drozdov I, Shalhoub J, Humphries J, Ladroue C, Didangelos A, Baumert M, Allen M, Davies AH, Monaco C (2011). Comparative lipidomics profiling of human atherosclerotic plaques. Circ Cardiovasc Genet.

[127] Cabanski M, Fields B, Boue S, Boukharov N, DeLeon H, Dror N, Geertz M, Guedj E, Iskandar A, Kogel U (2015). Transcriptional profiling and targeted proteomics reveals common molecular changes associated with cigarette smoke-induced lung emphysema development in five susceptible mouse strains. Inflamm Res.

[128] Hansen CS, Sheykhzade M, Moller P, Folkmann JK, Amtorp O, Jonassen T, Loft S (2007). Diesel exhaust particles induce endothelial dysfunction in apoE^−/−^ mice. Toxicol Appl Pharmacol.

[129] Folkmann JK, Risom L, Hansen CS, Loft S, Moller P (2007). Oxidatively damaged DNA and inflammation in the liver of dyslipidemic ApoE^−/−^ mice exposed to diesel exhaust particles. Toxicology.

[130] Cassee FR, Campbell A, Boere AJ, McLean SG, Duffin R, Krystek P, Gosens I, Miller MR (2012). The biological effects of subacute inhalation of diesel exhaust following addition of cerium oxide nanoparticles in atherosclerosis-prone mice. Environ Res.

[131] Poss J, Lorenz D, Werner C, Pavlikova V, Gensch C, Speer T, Alessandrini F, Berezowski V, Kuntz M, Mempel M (2013). Diesel exhaust particles impair endothelial progenitor cells, compromise endothelial integrity, reduce neoangiogenesis, and increase atherogenesis in mice. Cardiovasc Toxicol.

[132] Miller MR, McLean SG, Duffin R, Lawal AO, Araujo JA, Shaw CA, Mills NL, Donaldson K, Newby DE, Hadoke PW (2013). Diesel exhaust particulate increases the size and complexity of lesions in atherosclerotic mice. Part Fibre Toxicol.

[133] Lund AK, Knuckles TL, Obot Akata C, Shohet R, McDonald JD, Gigliotti A, Seagrave JC, Campen MJ (2007). Gasoline exhaust emissions induce vascular remodeling pathways involved in atherosclerosis. Toxicol Sci.

[134] Campen MJ, Lund AK, Knuckles TL, Conklin DJ, Bishop B, Young D, Seilkop S, Seagrave J, Reed MD, McDonald JD (2010). Inhaled diesel emissions alter atherosclerotic plaque composition in ApoE(^−/−^) mice. Toxicol Appl Pharmacol.

[135] Bai N, Kido T, Suzuki H, Yang G, Kavanagh TJ, Kaufman JD, Rosenfeld ME, van Breemen C, Eeden SF (2011). Changes in atherosclerotic plaques induced by inhalation of diesel exhaust. Atherosclerosis.

[136] Bai N, Kido T, Kavanagh TJ, Kaufman JD, Rosenfeld ME, van Breemen C, van Eeden SF (2011). Exposure to diesel exhaust up-regulates iNOS expression in ApoE knockout mice. Toxicol Appl Pharmacol.

[137] Bai N, Tranfield EM, Kavanagh TJ, Kaufman JD, Rosenfeld ME, van Eeden SF (2012). Exposure to diesel exhaust upregulates COX-2 expression in ApoE knockout mice. Inhal Toxicol.

[138] Campen M, Robertson S, Lund A, Lucero J, McDonald J (2014). Engine exhaust particulate and gas phase contributions to vascular toxicity. Inhal Toxicol.

[139] Vesterdal LK, Folkmann JK, Jacobsen NR, Sheykhzade M, Wallin H, Loft S, Moller P (2009). Modest vasomotor dysfunction induced by low doses of C60 fullerenes in apolipoprotein E knockout mice with different degree of atherosclerosis. Part Fibre Toxicol.

[140] Ross AF, Green WN, Hartman DS, Claudio T (1991). Efficiency of acetylcholine receptor subunit assembly and its regulation by cAMP. J Cell Biol.

[141] Chen LC, Nadziejko C (2005). Effects of subchronic exposures to concentrated ambient particles (CAPs) in mice. V. CAPs exacerbate aortic plaque development in hyperlipidemic mice. Inhal Toxicol.

[142] Sun Q, Wang A, Jin X, Natanzon A, Duquaine D, Brook RD, Aguinaldo JG, Fayad ZA, Fuster V, Lippmann M (2005). Long-term air pollution exposure and acceleration of atherosclerosis and vascular inflammation in an animal model. JAMA.

[143] Araujo JA, Barajas B, Kleinman M, Wang X, Bennett BJ, Gong KW, Navab M, Harkema J, Sioutas C, Lusis AJ, Nel AE (2008). Ambient particulate pollutants in the ultrafine range promote early atherosclerosis and systemic oxidative stress. Circ Res.

[144] Sun Q, Yue P, Kirk RI, Wang A, Moatti D, Jin X, Lu B, Schecter AD, Lippmann M, Gordon T (2008). Ambient air particulate matter exposure and tissue factor expression in atherosclerosis. Inhal Toxicol.

[145] Ying Z, Kampfrath T, Thurston G, Farrar B, Lippmann M, Wang A, Sun Q, Chen LC, Rajagopalan S (2009). Ambient particulates alter vascular function through induction of reactive oxygen and nitrogen species. Toxicol Sci.

[146] Quan C, Sun Q, Lippmann M, Chen LC (2010). Comparative effects of inhaled diesel exhaust and ambient fine particles on inflammation, atherosclerosis, and vascular dysfunction. Inhal Toxicol.

[147] Chen T, Jia G, Wei Y, Li J (2013). Beijing ambient particle exposure accelerates atherosclerosis in ApoE knockout mice. Toxicol Lett.

[148] Rao X, Zhong J, Maiseyeu A, Gopalakrishnan B, Villamena FA, Chen LC, Harkema JR, Sun Q, Rajagopalan S (2014). CD36-dependent 7-ketocholesterol accumulation in macrophages mediates progression of atherosclerosis in response to chronic air pollution exposure. Circ Res.

[149] Keebaugh AJ, Sioutas C, Pakbin P, Schauer JJ, Mendez LB, Kleinman MT (2015). Is atherosclerotic disease associated with organic components of ambient fine particles?. Sci Total Environ.

[150] Wan Q, Cui X, Shao J, Zhou F, Jia Y, Sun X, Zhao X, Chen Y, Diao J, Zhang L (2014). Beijing ambient particle exposure accelerates atherosclerosis in ApoE knockout mice by upregulating visfatin expression. Cell Stress Chaperones.

[151] Mauderly J, Kracko D, Brower J, Doyle-Eisele M, McDonald J, Lund A, Seilkop S (2014). The National Environmental Respiratory Center (NERC) experiment in multi-pollutant air quality health research: IV. Vascular effects of repeated inhalation exposure to a mixture of five inorganic gases. Inhal Toxicol.

[152] Yang Z, Knight CA, Mamerow MM, Vickers K, Penn A, Postlethwait EM, Ballinger SW (2004). Prenatal environmental tobacco smoke exposure promotes adult atherogenesis and mitochondrial damage in apolipoprotein E^−/−^ mice fed a chow diet. Circulation.

[153] Cakir Y, Yang Z, Knight CA, Pompilius M, Westbrook D, Bailey SM, Pinkerton KE, Ballinger SW (2007). Effect of alcohol and tobacco smoke on mtDNA damage and atherogenesis. Free Radic Biol Med.

